# Identification and management of frailty in English primary care: a qualitative study of national policy

**DOI:** 10.3399/bjgpopen20X101019

**Published:** 2020-03-18

**Authors:** Khulud Alharbi, Harm van Marwijk, David Reeves, Tom Blakeman

**Affiliations:** 1 PhD Student, Division of Population Health, Health Services Research & Primary Care, School of Health Sciences, University of Manchester, Manchester, UK; 2 GP and Professor of General Practice, Brighton and Sussex Medical School, University of Sussex, Brighton, UK; 3 Reader, National Institute for Health Research School for Primary Care Research, School of Health Services, University of Manchester, Manchester, UK; 4 Clinical Senior Lecturer in Primary Care, NIHR Collaboration for Leadership in Applied Health Research and Care Greater Manchester, Division of Population Health, Health Services Research & Primary Care, School of Health Sciences, University of Manchester, Manchester, UK

**Keywords:** frailty, general practice, normalisation process theory, policy

## Abstract

**Background:**

Policymakers are directing attention to addressing the needs of an ageing population. Since 2017, general practices in England have been contractually required to identify and code ‘frailty’ as a new clinical concept and, in doing so, support targeted management for this population with the aim of improving outcomes. However, embedding frailty policies into routine practice is not without challenges and little is currently known about the success of the programme.

**Aim:**

To explore the implementation of a national policy on frailty identification and management in English primary care.

**Design & setting:**

Qualitative study entailing interviews with primary care professionals in the North of England.

**Method:**

Semi-structured interviews were conducted with GPs (*n* = 10), nurses (*n* = 6), practice managers (*n* = 3), and health advisors (*n* = 3). Normalisation process theory (NPT) and ‘system thinking’ provided sensitising frameworks to support data collection and analysis.

**Results:**

Primary care professionals were starting to use the concept of frailty to structure care within practices and across organisations; however, there was widespread concern about the challenge of providing expanded care for the identified needs with existing resources. Concerns were also expressed around how best to identify the frail subpopulation and the limitations of current tools for this, and there was a professional reticence to use the term ‘frailty’ with patients.

**Conclusion:**

Findings suggests that additional, focused resources and the development of a stronger evidence base are essential to facilitate professional engagement in policies to improve the targeted coding and management of frailty in primary care.

## How this fits in

The ageing of the population is directing the attention of policymakers in England to help structure the targeted delivery of community care for patients with frailty. In England, improving the identification and management of frailty in primary care has recently become a requirement in the General Medical Services (GMS) contract. The authors of the present study considered the translation of this policy into routine practice. Analysis of professional accounts suggested a common concern about the challenge of providing care for the identified needs due to a shortage in resources. Without adequate resourcing, there is a high risk of bureaucratic implementation in which contractual requirements are met but without commensurate efforts to address unmet patient need.

## Introduction

The healthcare needs of an increasing number of older people pose challenges to health and social care systems worldwide.^1^ Along with this expansion in the ageing population comes an increasing need to address physical and mental frailty as a factor in planning person-centred care. In England, it is estimated that the overall prevalence of frailty is 14% and that prevalence increases with age, from 6.5% in those aged 60–69 years to 65% in those aged ≥90 years.^2^

Frailty has been defined as *’* *a multidimensional concept with dynamic interrelated factors in the physical, psychological, social, and environmental domains that affect the physiologic equilibrium of the person.* *’* ^3^ Patients with frailty are at higher risk of adverse events such as falls and hospitalisation.^4^ Frailty begins to develop at different points in adult life, and its stages differ among individuals depending on the care received, when they are diagnosed, and their socioeconomic context.^4,5^ Primary prevention has been proposed as a key strategy for minimising the costs of providing care to the ageing population,^6^ and this has drawn attention to the role of primary care professionals in planning and managing care for older adults by ensuring continuity and integration of care.^7^ However, to date, there has been no definitive marker for identifying frailty.^8^

In this context, the 2017/2018 GMS contract for England introduced a new requirement for general practices to identify and appropriately manage all patients aged ≥65 years with moderate or severe frailty.^9^ The long-term goal is to establish frailty assessment as an integral part of routine primary care practice and improve the ability of GPs to organise high-quality care for their more complex older patients, both within primary care and in collaboration with other services. As a first step, the scheme requires GPs to undertake routine frailty identification for all patients aged ≥65 years.^9^

NHS England has recommended use of the electronic frailty index (eFI) to aid identification of patients with frailty. The eFI automatically computes a ’frailty score’ based on the presence of up to 36 different health deficits using information extracted from a patient’s electronic health record and has been made available to all practices.^10^ This score is then classified into one of four levels of frailty (fit, mild, moderate, or severe) according to thresholds aligned with expected national prevalence rates (Table 1). However, policy guidance emphasises that the degree of frailty should not be based on the eFI alone; the final classification should be made only after clinical assessment with a tool such as the Clinical Frailty Scale,^9^ and possibly through discussion with patients.

**Table 1.** Electronic frailty index scores to define the categories in the electronic medical records^10^

**Category**	**Score, range**	**eFI development cohort, %**
Fit	0–0.12	50
Mild	0.13–0.24	35
Moderate	0.25–0.36	12
Severe	>0.36	3

eFI = electronic frailty index.

At this point in time, the contractual requirements around frailty have been in place for almost 2 years but it is unknown how far the ‘frailty agenda’ has impacted on primary care working practices, and there exists very limited evidence regarding the impact on health and social care organisation and outcomes. Recognising that all new GMS contract requirements have the potential to both structure and constrain care, the authors of this study used qualitative methods to understand the translation of this new policy into routine practice.^11^

## Method

### Study design

A framework approach was used as the strategy to guide the organisation and shaping of data.^12,13^ With a focus on understanding working practises within and across organisations, NPT^14–16^ and system thinking^17^ were used as sensitising devices to consider the different types of work being undertaken to implement the new frailty policy into routine practice.

### Sampling

Primary care professionals across the North of England were purposively sampled (Table 2). Primary care professionals directly involved in the implementation of the new frailty policy were invited for interview. To understand how implementation is being operationalised beyond these individuals, professionals working in the community but not directly involved in implementation of the new GP contract were also invited.

**Table 2.** Participant characteristics

	**GPs**(***n* = 10**)	**Nurses**(***n* = 6**)	**Practice managers**(***n* = 3**)	**Health advisors**(***n* = 3**)
**Age, years**				
31–40	2	0	1	2
41–50	4	2	1	1
>50	4	4	1	0
**Sex**				
Male	7	0	1	0
Female	3	6	2	3
**Region**				
Manchester	5	1	2	0
Yorkshire	2	4	1	3
North East	3	0	0	0
North West	0	1	0	0

#### ​Description of the sample

Twenty-two of 50 primary care professionals invited agreed to participate in the study. The final sample was spread across 10 practices and two community services, and comprised 10 GPs, six nurses, three practice managers, and three health advisors. Fourteen participants, including all GPs, practice managers, and one nurse were directly involved in implementation of the new contractual requirements within their practice. One of the GP participants also worked for the local clinical commissioning group (CCG). The remaining eight participants included five nurses and three health advisors based in the community.

The 10 practices involved in the study represented a wide range of practice sizes (median 10 175, range 4571–15 949), of which the median per cent of patients aged ≥65 years was 16.6% (range 4.77% to 28.87%).^18^ Area Index of Multiple Deprivation (IMD) scores also ranged widely, and included practices based in the most deprived 10% of local areas (first decile) up to the second most affluent 10% (ninth decile).^19^

### Data collection

Between March 2018 and August 2018, one author conducted semi-structured interviews at the participants’ workplaces. On average, the interviews lasted around 30 minutes (median 28 minutes, range 14–50 minutes). Interview topic guides were developed based on an a priori literature review in order to explore different types and levels of work relating to policy implementation (Box 1). NPT and its associated work-related constructs were also used as a lens to shape the topic guide. The interview schedule was pilot tested with two GPs to modify the interview questions. Emergent themes directed areas of questioning during subsequent rounds of data collection; this was carried out until no new themes emerged.

Box 1 **Interview topic guide**

**Table:** 

**Question areas**	**Prompts**
**Understanding the concept of frailty** Can I ask you what the term ’frailty’ means to you? Please describe the last patient you thought was clearly frail How do you usually use the word in your day? Are you aware about the requirements of the new GMS contract? What do you think about the requirements? Is it being enacted to meet older patient needs? To what extent do you think the new contract would help to address/improve frailty in populations? Are practice staff or community-based staff involved? How do people work together? In what ways? Can you give me an example of how the team works? What changes are you or your practice planning to make to meet these new requirements? What do you think are the key challenges to implement the new practice or manage frailty?	Do you view frailty as being a diagnosis or more of a process, like ageing? What made you think he or she was frail? How does this fit with what you want to do in terms of patient care? Can you give me an example of that? Are these tasks compatible with these people’s existing workload, skills, and professional identity? Are any informal care providers involved (for example, Age UK)? What is the capacity of general practices to do the work? Have new practice-wide policies been introduced? What do you think are the most important ways to overcome these challenges?
**Identifying frail people** How do you identify patients who are frail? How do you do your clinical assessment? Are you familiar with the eFI? (If ‘yes’: how is the eFI used in your daily practice or when you do your assessment at a patient’s home? If ‘no’: do you have a plan to use it in the future?) How do you respond when eFI pops up on your screen? If ignored, what are the reasons? What do you do then? Do you see benefits in identifying and coding frail patients as (a) derived by the patients, and/or (b) derived by the practice? Do you see any drawbacks?	Do you use standard instruments or your professional opinion? Is the same method used across the practice? Do you think the eFI will make a difference in terms of patient care, using resources, or minimising workload? Are you aware of the batch coding method? Does it help you to think about a patient in a different way? Which criteria does your practice use to justify the diagnosis? How do you document the diagnosis? What would you do with that code? How does it change what you do or could influence your decision?
**Managing frail people** How do you monitor the care of your patients with frailty? How do you describe the system you work in? How would you analyse and diagnose where the system can be improved? What are the challenges in the system to manage frailty?	What type of system do you use to track and follow these patients? Describe the process staff use to work together to care for patients with frailty? What are the different systems your system interacts with, and how does your system interact with these systems? How can we manage these challenges?
**General** Do you have any final comments or suggestions concerning the care of frail patients in primary care?	Is there anything you want to say that we have not yet discussed?

eFI = electronic frailty index. GMS = General Medical Services.

### Data analysis

Audio-recordings were professionally transcribed verbatim. Analysis was based on the constant comparative method, along with open, focused, and theoretical coding to analyse the data.^20^ The interviewing author developed an initial set of codes using in vivo coding, coded each interview transcript individually, and then compared the old and new codes.^20^ As a team, the authors immersed themselves in the data to let the codes, and then the categories, emerge inductively. The interviewing author then charted the data into the matrix, which was used to compare and contrast within and across participants’ accounts. The authors then developed analytic memos to explore the implementation processes within each practice, which enhanced the ability to extract differences and similarities across practices. A coding framework, informed by NPT,^14–16,21^ was designed that related to the core constructs of the theory (Box 2) and so identified and delineated the different types of frailty-related work undertaken in primary care. Such findings were compared and contrasted between these levels of the analysis in order to increase their consideration from a practical point of view.

Box 2 **Normalisation process theory: a sensitising framework for understanding the work surrounding the frailty policy in primary care^14–16,21^**

**Table:** 

**Coherence**(**sense-making work**)	**Cognitive participation**(**relationship work**)	**Collective action**(**enacting work**)	**Reflexive monitoring**(**appraising work**)
**Differentiation**	**Enrolment**	**Interactional workability**	**Reconfiguration**
Defining, dividing up, and categorising task: What do participants think of the concept ’frailty care’ and their experiences delivering it? What do participants think about frailty and its relevance to their work?	Recruiting the self and others to tasks: Do participants believe they are the correct people to drive the implementation forward? Do participants engage with other staff within or across organisations to implement the frailty policy? Who initiates the engagement? Who does and who does not ‘buy-in’ to implement the frailty policy?	Doing tasks, and making outcomes, in practice: How is the term ‘frailty’ discussed in consultations? How do the new requirements affect discussions between patients and professionals? Does implementing the frailty concept make it easier or harder to identify the patient?	Changing tasks: Has identifying frailty been adapted based on experience? If so, how?
**Individual specification**	**Initiation**	**Relational integration**	**Individual appraisal**
Making sense of personal versions of tasks: Are the requirements in the new contract easy to implement? Do participants understand what tasks/practice require of them? Do the new requirements bring any benefits?	Organising an individual contribution to tasks: Who actively engages to plan/prepare working with a new contract? Are participants prepared to work with a new contract? Are individuals prepared to invest time, energy, and work into a particular practice? If so, what is this work?	Making and communicating reliable knowledge about tasks: How do the new requirements (such as, identifying frailty) affect trust and confidence between patients and professionals, or between different groups of professionals? How do professionals work to enact new contracts and maintain relationships?	Individual evaluation of contributions and tasks: Is it clear what effects a particular practice (such as, identifying frailty) has had? Do individuals make efforts to reflect on/appraise work around frailty? If so, how? Has appraisal work informed whether a particular practice around frailty is advantageous for patients and staff?
**Communal specification**	**Activation**	**Skill set workability**	**Communal appraisal**
Making sense of shared versions of tasks: Who does/does not think implementing a frailty concept is a good idea? Are the benefits of a particular practice/task (such as, identifying frailty) valued by all participants? Does a particular task fit with the overall goals and activity of the practice?	Organising a shared contribution to tasks: Whether the participants can undertake their roles and tasks, whether any barriers and facilitators are encountered to delivering care for patients with frailty based on the contract How does a particular task/practice (such as, identifying frailty) feature in practice meetings? Does the practice team undertake work to arrange a shared contribution to implement frailty policy? If so, what is this work?	Allocating tasks and performances: What impact does the introduction of the new contract have on responsibility? How is a particular frailty requirement distributed within the practice team? Is the work being devolved to others? If so, how and for what reason? Does the introduction of identifying frailty alter the awareness of the work done by other members within a practice team?	Shared evaluation of contributions and tasks: Do participants contribute or share feedback about a particular practice (such as, identifying frailty) with others? If so, what is discussed? Has appraisal work informed whether a particular practice of frailty policy is advantageous for patients and staff?
**Internalisation**	**Legitimation**	**Contextual integration**	**Systematisation**
Learning how to do tasks in context: Has there been an understanding of how to implement the new requirement? Does the staff have time to learn to understand and carry out the new policy?	Making tasks the right thing to do: Do the participants believe it is appropriate for them to be involved in the new contract/requirements?	Supporting and resourcing tasks in their social context: How is the new contract linked to organisational structure (such as, practice meetings and using guidance)? Do the participants support frailty policy in all important ways? Are they capable of implementing the new contract? How is a particular task (such as, identifying frailty) resourced?	Organising a reliable stock of knowledge about tasks: Has the organisation developed ways of keeping up to date with approaches to managing a set of practices (such as, the management of frailty)?

The principles of system thinking were used to frame team discussions and to encourage development of categories, improve data analysis, and consider implementation issues around frailty from different perspectives and experiences.^17^ NVivo (version 11) provided a structure to support qualitative data analysis. The authors met weekly for around 8 months to interpret the data, compare themes, resolve discrepancies, and clarify meanings. The analysis continued iteratively, with the resulting categories centred around three key sets of practices concerning frailty: (1) bringing frailty into view; (2) identifying frailty; and (3) managing frailty.

## Results

A core theme raised by interviewees was the challenge in navigating the relationship between supply and demand generated by adding consideration and management of frailty into routine daily practice. Access to, and the mobilisation of, resources were key factors influencing professional engagement to focus on the identification and management of patients with frailty in routine practice. From analysis, the authors organised the views of participants around the work engendered by the new policy into three key areas: (1) bringing frailty into the view of primary care professionals; (2) the identification and coding of patients with frailty; and (3) the subsequent management of those patients.

### Bringing frailty into view

Accounts suggested that the concept of frailty was beginning to inform the delivery of care within practices as well as across organisations. For some participants, giving attention to frailty offered the potential to redirect resources:

*​‘Yes. It’s obviously one thing leads to another, so if you put the resources in place, if you identify what their healthcare needs are for the patient then the inevitable consequence is better outcomes.’* (Participant [P]3, GP)

*​‘*… *it’s a very hot topic at the minute, so there are lots of people talking about it, almost making it a sexy topic, which is great, because then you get people buying in with it, finances follow, resources follow, so that's great, so it's really I suppose the important thing is to seize the opportunity now before something else comes along and takes over or it fades in the background.’* (P7, GP)

Within routine practice, some GPs and nurses described that flagging up frailty could help inform decision-making with patients about treatment options as well as encourage broader conversations to consider both medical and social needs. This was also seen as providing an alert to trigger a lower threshold for clinical review during episodes or acute illness:

*​‘*... *it will help us think of them and perhaps have a look to the fact that there is something on the radar that is making them appear frail;* ... *whether they can be safely managed at home or whether I need to think about anything else when they’re acutely unwell, as to if you were asking the questions, have you got family or somebody else who was available to help or lives close by;* … *’* (P17, GP)

Nonetheless, most participants expressed concerns that the introduction of frailty into the contract was bringing previously unidentified needs to the surface but that there was currently insufficient capacity in primary care and community services to manage these newly identified needs. There was a common suggestion that achievement of the planned outcomes may require additional resources:

*​‘But identifying it as a priority isn’t the solution. That’s the start* … *with anything like this, what you’re likely to do if you actually look at a group of people who aren’t having all their needs met, then you’re going to find other needs. So you’re going to end up needing, in the short-term, more resources. And they never kind of seem to see that bit. It’s always about, well we can do this and that, we’ll keep people out of hospital and it will save money.’* (P2, GP)

This concern over the potential increased demand on resources was somewhat at odds with a widespread and somewhat cynical view that the underlying principal aim of the policy was really to reduce the costs associated with unplanned hospitalisation:

*​‘So, I don’t think it actually necessarily is about preventing or keeping them alive longer, I think it’s more about keeping them out of hospital.’* (P13, GP)

### Identifying frailty

Rather than viewing frailty as an entirely new approach to care stratification, participants considered it to relate to the previous contractual requirements that focused on identifying and planning care for the most vulnerable 2% of patients at risk of unplanned hospitalisation.^22^ As such, from a contractual perspective, the frailty policy was seen as relatively straightforward to fit into their daily practice:

*​ ‘We've just now given them another label. We were still managing them beforehand and doing things with them. It's just that we've just added another label into the system.’* (P15, GP)

Some participants described their experiences with the eFI and how it might help to increase their awareness of patients where frailty was a relevant consideration:

*​‘I think it is quite good because I think it’s quite broad and it’s based on quite good information. I think the main good part of it is for case finding more than anything* … *it’s not perfect, but it is quite good to pick up new patients.’* (P14, GP)

*​‘So, it’s really good, in that you can use it in a proactive way, to look at how we can minimise some of the harm that’s happening, some of the risks, things like falls, keeping people well and active.’* (P20, nurse)

However, there was also a degree of uncertainty around the use of the eFI in terms of its evidence base and its use as an approach for identifying patients with frailty. Some participants reported adapting their approach through the use of additional or alternative ways of targeting patients most likely to be frail:

*​‘*... *as a community matron, we’ve used all sorts of tools in the past to try and identify that top per cent of the triangle, and none of them have really been truly effective* ... *we’re going to look at and target a particular area where there’s a lot of older people. And then go in and see that group of people who live in those bungalows, in that social housing, in that sheltered accommodation that we review all the patients who are in there. As opposed to looking at a frailty index.’* (P6, nurse)

Doubt over the eFI was expressed in terms of understanding the underpinning algorithm and how the scores were calculated for each patient:

*​‘But unfortunately, most the clinicians that I spoke to, they don’t understand how the algorithm’s got to that score in the first place* … *I think it’s a real shame that there’s so much hidden behind that score, that people don’t really understand it. And even if you’ve explained it, it’s quite complex. It’s difficult for people to retain.’* (P12, practice manager)

Participants felt that a key problem with the eFI was its dependency on historical data stored in the patient’s computerised records, which could be old, variable, inaccurate, or missing relevant information:

*​‘And my experience of coding from practice to practice, some practices are really, really good at the coding, and others aren’t. So, you’ve got to bear that in mind.’* (P21, nurse)

*​‘For example, certain categories like polypharmacy or falls or that sort of thing, that's how it generates the frailty score. If those things aren't coded it's not going to pick them up when you run the program and therefore the score will be inaccurate.’* (P7, GP)

Moreover, participants raised concerns that the eFI does not necessarily capture the severity of either a condition or a functional deficit. Nor was it seen to account for a person’s coping ability or social circumstances:

*​‘I don’t think it’s great actually because there’s some* … *the options we get for* … *for say like continence* … *according to the computer, you’re either continent or you’re incontinent. There’s no kind of in between and it could be, you know, somebody’s slightly incontinent because they’ve got arthritis and they can’t get to the toilet in time, but that might be just once every couple of days that they’re just being a little bit incontinent.’* (P5, nurse)

As specified in the contract itself, participants tended to consider that ‘applying clinical judgment’ was essential before making a final decision on an individual patient’s degree of frailty before coding.^9^ However, despite this and the concerns about the validity of the eFI score, participants’ accounts also suggested that this step was not always followed:

*​‘However, there has been a process called batch coding which started to go on, which I picked up on. Batch coding by* […] *It’s a method on the computer where they can find, like, search all the electronic frailty indexes and change them into diagnoses on the computer. And this has been going, and is probably still going on, despite the department actually asking us not to do it. I wouldn’t be surprised if it’s still going on. And once it gets on a patient’s records it can impact on lots of other things.’* (P4, GP)

### Managing frailty

Analysis of participants’ accounts suggested there was a spectrum of approaches to the implementation of the frailty policy, from bureaucratic (‘batch coding’) implementation to organisations developing asset-based, bottom-up approaches. Though the contract was seen to have brought frailty into view, some participants questioned the effectiveness of the contractual requirements in isolation. Accounts suggested it was the availability and mobilisation of resources within practices and across services that were more likely to be drivers of change:

*​‘*... *so, the contract is almost something you have to tick the box to be compliant with your contract but I don’t think it adds much to the patient care, would be my opinion.’* (P11, GP)

*​‘*… *but it’s just how do you shift some of the resource between being reactive, to being proactive. So, sometimes I think it’s more than a contract isn’t it, it’s a way of thinking, and it’s a way of organisations, how services are commissioned. There are bigger things at play, aren’t there, rather than just a contract.’* (P20, nurse)

A balancing act was often described with regard to maximising the utility of frailty as a concept to improve care and outcomes, while at the same time minimising the potential for burden in terms of labelling a patient as frail. Overall, there was reticence to use of the term ‘frailty’ with patients during clinical encounters. Suggested terminology to address this concern included referring to a patient’s Rockwood^23^ score:

*​‘I just don’t think it comes up in conversation. You know, I wouldn’t look at their record and then say to them, well I see you’re frail or, you know, how do you live with frailty, you know I’m quite sensitive about using the word frailty to people* ... *’.* (P19, health advisor)

*​‘If you just wrote the Rockwood score in, rather than the word frail* … *I think that word ”frail” is not nice* ... *’.* (P4, GP)

There was evidence of delegation and distribution of work within practices and across organisations to implement the frailty agenda. As examples, these included the role of healthcare assistants to ask about falls during routine review appointments and then direct patients to the practice community matron for further assessment if necessary. In another locality, an integrated system with a care coordinator to improve connections between services and patients as well as manage the workload across practices was implemented. For some, pharmacists had become involved:

*​‘Right, we’ve only just started looking at the frailty as such, as in a policy. I’ve written a policy, and I asked the community pharmacist to have a look at it, the CCG pharmacist. She’s put some input on the medication.’* (P16, nurse)

For most participants the frailty agenda had influenced multidisciplinary care, including the establishment of meetings. One model involved six practices and a community team who were seeking to develop a frailty clinic. The aim was to allocate more time to patients with frailty and, at the same time, to take the workload outside of the GP clinic. However, managing the demand still remained a key issue:

*​‘So it’s trying to get them in, seeing everybody in the same setting, and getting that patient sorted then and there. So it’s trying to give them a better service but it’s trying to release more time for us as well so then hopefully we will reduce the demand and then hopefully we’ll be able to say okay, actually we can offer longer appointments, but with the demand that we have we can’t. So you get yourself in a bit of a catch 22.’* (P14, GP)

Concerns were expressed regarding the sustainability of new projects focused on frailty management and the resource dependency required even in areas where evaluation has demonstrated positive effects:

*​‘They’re trying to make out we’re getting lots of support but we’re not. Not in every area. Maybe in the pilots there’s good support but as soon as a pilot’s been proven everybody forgets about the support which was put in during the pilot and it’s left on the GPs doorstep.’* (P4, GP)

*​‘*… *so, there’s a big feeling, in the GP world anyway, that that shift of funding doesn’t need to be huge, but it needs to happen.’* (P1, practice manager)

## Discussion

### Summary

This study highlights tensions experienced by professionals surrounding the recent introduction of the routine identification and management of patients with frailty in general practice. There was evidence that the concept of frailty was broadly welcomed, and beginning to inform and structure care within practices and across organisations; however, while the identification and coding of patients with frailty was seen to offer opportunities to guide a broader approach to health and social care delivery, concerns were expressed around the utility of the eFI as a tool for case finding. Participants considered that identifying and assessing all their patients with frailty has the potential to uncover considerable unmet need, and there was widespread concern over the availability of resources to meet the resulting increased demand on services.

### Strengths and limitations

Use of qualitative methods enabled an early understanding of the implementation of frailty policies into routine practice. The authors acknowledge that the findings have been constructed through interaction with the data and participants. A number of methods were used in order to enhance the trustworthiness of the study and to comprehensively analyse the data.^12,24^ The analysis process was inductive where open coding took place, and then deductive through use of the NPT framework.^14–16^ The authors then compared the findings and contrasted between these levels of the analysis in order to abstract the main theme and then the subthemes. Furthermore, system thinking provided sensitising device to explore working practices and enhance the level of discussion during research meetings.^17^ The present study primarily focused on implementation of the frailty agenda within the context of the GP contract;^9^ however, sampling professionals working in social care may have further enhanced understanding of the broader management of people with frailty in the community.

Resonating with previous research, participants expressed a reluctance to use the term ‘frailty’ in communication with patients.^25^ Future ethnographic research entailing observation as well as interviews with patients is necessary to explore the framing of frailty care within consultations and across systems of care. Professionals participating in the study may have had a greater interest in frailty; however, analysis still highlighted tensions underpinning the introduction of frailty into the GP contract. Analysis of accounts suggested a range of approaches to implementation, including the potential for ‘batch coding’ of patients identified through use of the eFI. Further quantitative evaluation is needed to examine the approaches being undertaken.

### Comparison with existing literature

Prior studies have noted that the eFI may help clinicians to plan care taking into account an individual’s degree of frailty,^23^ and support allocation of community services for the population of people with frailty.^26^ However, participants’ accounts suggested a need for a greater clarity on the evidence supporting its use, including better understanding of the algorithm underpinning the tool. In terms of accuracy, a recent study has indicated that the eFI might correlate more highly with other frailty scales by including more functional items.^27^ Research has also shown a strong association between a patient’s eFI score and length of registration at their practice, as the tool searches across the full record and all deficit codes are treated as non-resolvable, including conditions such as UTIs, peptic ulcer, and depression.^28^

Since 2017, the GP contract has focused on the identification of frailty in patients aged ≥65 years;^9^ however, a key epidemiological study by Barnett et al (2012) highlighted that the onset of multimorbidity occurs 10–15 years earlier in areas of socioeconomic deprivation.^29^ As such, research is required to ensure that frailty policies and interventions do not reinforce the inverse care law and further disadvantage frailty in younger age groups in more socioeconomically challenged populations.^30^

### Implications for research and practice

Achievement of the full patient benefits envisaged under the frailty agenda is likely to require a major resource reallocation. These findings resonate with the British Medical Association’s report, *Investment in* *G* *eneral* *P* *ractice in England*,^31^ as well as research by Hobbs *et al*, which indicates that GP workload is nearing *‘* *saturation point.* *’* ^32^As has been shown in the implementation of previous policies relating to the organisation of care for people with long-term conditions, there is a risk that the frailty contractual requirements will be achieved but only in a bureaucratic manner.^33^ The 2019–2020 GP contract focuses on the establishment of primary care networks and some investment in a wider workforce, which may be a starting point to address this challenge.^34^

Buy-in to this approach to structuring care will also depend upon healthcare professionals perceiving direct benefits for their patients; however, current evidence for the effectiveness of interventions to prevent adverse outcomes in patients with frailty is weak at best,^35^ with possible exceptions for certain subgroups.^36^ A recent systematic review of comprehensive geriatric assessments in primary care also reported only limited benefits for patients.^37^ NHS England have recommended further research based on the potential benefits for some critical outcomes,^38^ but we are currently far from having a clear insight into approaches that work and for whom.

The present study analysis underlines the challenges of implementing a new policy in a complex health system where design, implementation, and support all involve different sets of actors with different understandings and levels of capacity and resources. This study highlights that GPs are contractually tasked to address frailty pressures within the NHS but have concerns over taking on considerable additional workload without additional resources.

In summary, this qualitative study suggests that primary care professionals have broadly welcomed the introduction of frailty assessment and management as an integral part of their daily practice, and see the potential for this to help inform and structure the care they deliver for an ageing population. However, additional resources and development of a stronger evidence base will be essential in order to achieve the desired improvements in care and outcomes. Without this, there is a high risk of bureaucratic implementation in which contractual requirements are met but without commensurate efforts to address unmet patient need.
